# Role of distinct type‐IV‐secretion systems and secreted effector sets in host adaptation by pathogenic *Bartonella* species

**DOI:** 10.1111/cmi.13004

**Published:** 2019-02-06

**Authors:** Alexander Wagner, Christoph Dehio

**Affiliations:** ^1^ Focal Area Infection Biology, Biozentrum University of Basel Basel Switzerland

**Keywords:** *Bartonella*, Bep effector proteins, host adaptation, Trw, type‐IV‐secretion systems, VirB/VirD4

## Abstract

The α‐proteobacterial genus *Bartonella* comprises a large number of facultative intracellular pathogens that share a common lifestyle hallmarked by hemotrophic infection and arthropod transmission. Speciation in the four deep‐branching lineages (L1–L4) occurred by host adaptation facilitating the establishment of long lasting bacteraemia in specific mammalian reservoir host(s). Two distinct type‐IV‐secretion systems (T4SSs) acquired horizontally by different *Bartonella* lineages mediate essential host interactions during infection and represent key innovations for host adaptation. The Trw‐T4SS confined to the species‐rich L4 mediates host‐specific erythrocyte infection and likely has functionally replaced flagella as ancestral virulence factors implicated in erythrocyte colonisation by *bartonellae* of the other lineages. The VirB/VirD4‐T4SS translocates *Bartonella* effector proteins (Bep) into various host cell types to modulate diverse cellular and innate immune functions involved in systemic spreading of bacteria following intradermal inoculation. Independent acquisition of the *virB/virD4/bep* locus by L1, L3, and L4 was likely driven by arthropod vectors associated with intradermal inoculation of bacteria rather than facilitating direct access to blood. Subsequently, adaptation to colonise specific niches in the new host has shaped the evolution of complex species‐specific Bep repertoires. This diversification of the virulence factor repertoire of *Bartonella* spp. represents a remarkable example for parallel evolution of host adaptation.

## 1 INTRODUCTION

**Figure 1:**
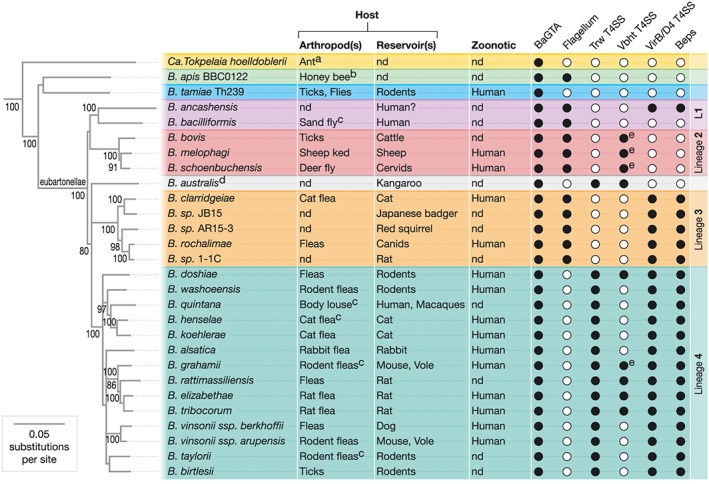
Phylogeny of *Bartonella* and distribution of key virulence factors. Phylogeny of the genus *Bartonella* with the ant‐specific species (a) *Candidatus* Tokpelaia hoelldoblerii as outgroup taxon. The phylogenetic pattern resembles the tree topology from (Segers, Kesnerova, Kosoy, & Engel, 2017) and shows the three *Bartonella* clades composed of the honeybee symbiont (b) *Bartonella apis*, pathogenic *Bartonella tamiae*, and the eubartonellae. Eubartonellae are further separated into four lineages and Bartonella australis (d). The phylogenetic tree was inferred based on a concatenated alignment of five core protein sequences. Indicated are arthropod (C‐confirmed vectors) and reservoir hosts, as well as the zoonotic potential of *Bartonella* spp. The presence and absence of key virulence factors is indicated by full and empty circles, respectively. In contrast to chromosomally encoded VbhT T4SSs, the plasmid encoded counterparts are indicated with an (e) next to the full circle. BaGTA: *Bartonella* gene transfer agent; T4SS: type‐IV‐secretion system; Bep: *Bartonella* effector protein; nd: not determined

The α‐proteobacterial genus *Bartonella* comprises a vast number of facultative intracellular pathogens that cause acute and chronic infections at high prevalence in a broad spectrum of mammals including humans. Transmission of *Bartonella* spp. is mediated by diverse hematophagous arthropod vectors, in which the bacteria typically colonise the midgut. Experimentally proven vector competence was demonstrated thus far only for five *Bartonella* species: *Bartonella bacilliformis* (sand fly), *Bartonella quintana* (human body louse), *Bartonella henselae* (cat flea), and Bartonella grahamii and *Bartonella taylorii* (both via rodent fleas; Bown, Bennet, & Begon, 2004; Byam & Lloyd, 1920; Hertig, 1942; Koehler, Glaser, & Tappero, 1994; Figure 1). However, various other fleas, lice, sand flies, keds, mites, and ticks may represent competent vectors based on the frequent detection of *Bartonella*‐DNA in these blood‐sucking arthropods (Iannino, Salucci, Di Provvido, Paolini, & Ruggieri, 2018). Recent phylogenetic analyses shed light on the evolutionary history of the genus *Bartonella* by identifying ancestral Bartonellaceae that inhabit the gut of various ant species as nutritional symbionts (Bisch et al., 2018; Neuvonen et al., 2016). Furthermore, the honeybee gut symbiont *Bartonella apis* was shown to form a monophyletic clade with human pathogenic *Bartonella tamiae*, that diverged before the monophyletic group of the eubartonellae formed by radiation of four deep‐branching lineages (L1–L4; Kesnerova, Moritz, & Engel, 2016). The eubartonellae displaying a common hemotrophic lifestyle and transmission by blood‐sucking arthropods have thus evolved from insect gut symbionts (Bisch et al., 2018; Segers et al., 2017).

*Bartonella* spp. are highly adapted to one or few mammalian reservoir hosts, where they cause long‐lasting bacteremia. Species confined to L2 exclusively infect ruminants, such as deer‐specific *Bartonella schoenbuchensis* or the cattle‐specific *Bartonella bovis*. In contrast, representatives of the species‐rich L3 and L4 infect a wide variety of mammalian reservoir hosts. Examples of parallel adaptation to the same reservoir hosts are described for rats (L3: *B. sp*.1‐1C; L4: *Bartonella tribocorum*), dogs (L3: *Bartonella rochalimae*; L4: *Bartonella vinsonii berkhoffii*), and cats (L3: *Bartonella clarridgeiae*; L4: *B. henselae*; Engel et al., 2011; Harms, Segers, et al., 2017b; Figure 1). Reflecting various levels of host adaptation in the reservoir host, *Bartonella* infections manifest by a broad spectrum of symptoms. These range from subclinical courses (many animal‐specific species) to moderate morbidity diseases (such as human trench fever by the L4 human‐species *B. quintana*) to life threatening disease exemplified by Carrion's disease caused by the human‐specific L1‐species *B. bacilliformis* (Gomes & Ruiz, 2018). Of note, the recently described *Bartonella ancashensis* (L1) was isolated from patients diagnosed for Carrion's disease, indicating that this species can also cause disease symptoms similar to the closely related *B. bacilliformis* (Hang et al., 2015).

Incidental transmission of animal‐specific *Bartonella* spp. to humans as non‐reservoir host can lead to zoonotic disease associated with a broad range of clinical manifestations, such as fever, lymphadenopathy, neuroretinitis, endocarditis, encephalitis, and myocarditis. The best‐characterised zoonotic pathogen is the cat‐specific L4‐species *B. henselae*, which causes the majority of *Bartonella* infections worldwide, including cat scratch disease in immunocompetent patients and bacillary angiomatosis or peliosis in immunocompromised patients (Florin, Zaoutis, & Zaoutis, 2008). More and more *Bartonella* species are recognised as zoonotic pathogens causing infections of high worldwide prevalence (Gomes & Ruiz, 2018; Iannino et al., 2018; Figure 1).

Among eubartonellae, the life cycle and infection strategy is best studied for L4 species (Koesling, Aebischer, Falch, Schulein, & Dehio, 2001), but it is believed that the general concept of reservoir host infection is shared by all eubartonellae (Siamer & Dehio, 2015). Bacteria colonising the arthropod midgut are shed with the faeces onto the mammalian skin and are superficially inoculated into the derma by scratching or biting (Chomel et al., 2009). In the “dermal niche” bacteria may colonise migratory immune cells such as dendritic cells, before they spread to and colonise the “blood‐seeding niche” that is considered to include endothelial cells (Okujava et al., 2014). Transmission from the dermal niche to the blood seeding niche may occur via the lymphatic system (Hong et al., 2017; Okujava et al., 2014). From the blood‐seeding niche bacteria are periodically released into the bloodstream, where they invade, replicate, and persist within erythrocytes (Okujava et al., 2014; Scherer, DeBuron‐Connors, & Minnick, 1993; Schulein et al., 2001; Vieira‐Damiani et al., 2016). Periodic seeding into blood ceases with the establishment of an antibody titre against *Bartonella*, while already intraerythrocytic bacteria are protected from antibodies or any other immune response allowing their persistence in circulating blood for the remaining life‐span of the colonised erythrocyte (Koesling et al., 2001). The resulting long‐lasting intraerythrocytic bacteremia represents a specific adaptation to the mode of transmission by blood‐sucking arthropods. A blood meal by a competent arthropod vector thus closes the infection cycle. The full infection cycle occurs per definition exclusively within reservoir hosts and competent arthropod vectors, whereas it may differ at least in parts within incidental hosts.

The pathogenicity of *Bartonella* spp. relies on a multitude of virulence factors (e.g., flagella, hemin‐binding proteins, and trimeric autotransporter adhesins such as BadA or Vomp) that are crucial at certain stages of the infection cycle (Harms & Dehio, 2012; Saenz et al., 2007; Vayssier‐Taussat et al., 2010). A hallmark of the molecular pathogenicity of *Bartonella* infection, however, is the involvement of distinct type‐IV‐secretion systems (T4SSs) in erythrocyte invasion (Trw‐T4SS; Vayssier‐Taussat et al., 2010) and in the subversion of cellular functions of other target cells, for example, dendritic cells, macrophages, and endothelial cells (VirB/VirD4‐T4SS; Schmid et al., 2004; Schulein & Dehio, 2002). Although the VirB/VirD4‐T4SS translocates *Bartonella* effector proteins (Beps) in order to enable the bacteria to reach and colonise the blood‐seeding niche, the Trw‐T4SS does not translocate any effector, but instead mediates adhesion to erythrocytes via surface‐exposed pili.

In this review, we will focus on the adaptive evolution of the distinct T4SSs and Beps and their role during *Bartonella* infection. We will furthermore discuss the contribution of these virulence factors to host adaptation and the resulting remarkable degree of host specificity observed among eubartonellae.

## 2 ACQUISITION OF THE CONJUGATIVE T4SS VBH

The acquisition, expansion, and functional diversification of *Bartonella*‐specific virulence factors (that is, absent in *B. apis* and *B. tamiae,* but present in eubartonellae) enabled rapid host switches that led to the explosive radiation within the eubartonellae (Engel et al., 2011; Segers et al., 2017). Among these *Bartonella*‐specific virulence factors, T4SSs are best characterised. T4SSs are macromolecular machines that mediate the interbacterial transfer of a nucleoprotein complex (relaxase‐ssDNA) in a process known as bacterial conjugation and the interkingdom translocation of effectors from bacteria into eukaryotic host cells (Grohmann & Christie, 2018). Genomes of all eubartonellae, except *B. bacilliformis* (L1), encode one to three distinct T4SSs: Trw, Vbh/TraG, and VirB/VirD4 (Figure 1).

The Vbh (VirB homologous) T4SSs and associated TraG T4CP (Type IV secretion coupling protein) are encoded on plasmids or chromosomally by *Bartonella* spp. (Figure 1). As the sole T4S‐machinery present in L2, and due to its genomic link to a toxin (VbhT) resembling the Beps, the VbhT/TraG‐T4SS has been proposed to play a role in pathogenicity (Harms & Dehio, 2012). However, a recent study showed that the plasmid‐encoded Vbh/TraG‐T4SS of *B. schoenbuchensis* plasmid pVbh functions as a classical conjugation system (Harms, Liesch, et al., 2017a). Importantly, VbhT represents a second substrate (next to the relaxase‐ssDNA substrate) translocated into recipient bacteria, thus representing an interbacterial effector rather than an interkingdom effector as suggested earlier. VbhT was shown to inactivate type II topoisomerases (gyrase and topoIV) by covalent modification, but the biological role of the resulting changes in DNA topology in recipient cells remains unknown (Harms et al., 2015; Harms, Liesch, et al., 2017a). In contrast to the plasmid‐encoded *vbh/traG* loci in L2, the majority of their chromosomally encoded counterparts present in some L4 species typically contain deleterious mutations and lack *traG*/*traA* genes encoding the crucial accessory components for conjugation. The chromosomally encoded Vbh‐T4SSs thus represent remnants of a deteriorating conjugation system (Harms, Liesch, et al., 2017a).

## 3 ACQUISITION OF THE TRW T4SS MEDIATING HOST‐SPECIFIC ERYTHROCYTE ADHESION

The presence of the Trw‐T4SS is restricted to Bartonella australis and to L4‐species (Figure 1), in which it mediates host‐specific adhesion to erythrocytes (Deng, Le Rhun, Le Naour, Bonnet, & Vayssier‐Taussat, 2012; Vayssier‐Taussat et al., 2010). This *bona fide* virulence factor is ancestrally related to the enterobacterial Trw conjugation system encoded by the conjugative plasmid R388. Following horizontal acquisition the *trw* locus was integrated into the bacterial chromosome. Notably, acquisition of the Trw‐T4SS coincided with loss of flagella, which are known to contribute to erythrocyte infection by *B. bacilliformis*, and likely also by species of L2 and L3 (Harms & Dehio, 2012). Functional replacement of flagella by the Trw‐T4SS in L4 may have been driven by an increased capacity for host adaptation as exemplified by association with the most extensive adaptive radiation within the eubartonellae (Harms & Dehio, 2012).

In contrast to the other T4SSs present in the Bartonellaceae, the Trw system does not encode a T4CP, which is crucial for substrate translocation. Indeed, species‐specific erythrocyte infection by the Trw‐T4SS is not reliant on effector translocation, but on the extracellular exposure of variable pilin subunits (i.e., TrwL and TrwJ; Deng et al., 2012; Vayssier‐Taussat et al., 2010). For instance, a TrwJ paralogue from the mouse‐specific pathogen *Bartonella birtlesii* has the ability to bind to mouse erythrocytes, but not to cat erythrocytes. It was further shown that a TrwJ paralogue binds the major glycoprotein band3 at the surface of erythrocytes (Deng et al., 2012). The Trw‐T4SSs encode multiple variant copies of pilin subunits, which are the result of gene duplication and diversification events. It is thus conceivable that the polymorph surfaces of erythrocytes were the driving force of this pilin diversification (Harms & Dehio, 2012). In summary, the Trw‐T4SS is a key virulence factor mediating reservoir‐host‐specific erythrocyte infection by L4 *Bartonella* and likely B. australis and appears to have played a pivotal role in adaptation to new mammalian hosts.

## 4 LINEAGE‐SPECIFIC ACQUISITION OF THE PROTEIN EFFECTOR‐TRANSLOCATING T4SS VIRB/VIRD4

To date, the best characterised *Bartonella*‐specific virulence factors are the VirB/VirD4‐T4SS and the arsenal of translocated Beps. Multiple studies have established their diverse roles in modulation of diverse cellular functions in nucleated mammalian cells in vitro and their importance for reaching and colonialization of the blood‐seeding niche in vivo (Harms & Dehio, 2012). Recent phylogenetic and genomic analyses revealed that the *virB/virD4*‐T4SS loci including a primordial *bep* gene were acquired at three occasions independently within the eubartonellae (Engel et al., 2011; Harms, Segers, et al., 2017b). They have been identified in all L3 and L4 species and in the recently discovered *B. ancashensis* (L1); however, they are absent from *B. bacilliformis* (L1) and all L2 species (Harms, Segers, et al., 2017b; Figure 1).

Interestingly, the distributional pattern of VirB/VirD4‐T4SSs and Beps correlates with the blood‐feeding behaviour and proposed mode of transmission by the arthropod vectors considered competent for bacterial transmission (Dehio & Tsolis, 2017). Bartonellae lacking a VirB/VirD4‐T4SS (i.e., *B. bacilliformis* and L2 species) are exclusively transmitted by biting diptera (sandflies, biting flies, or keds) that display a forceful mode of blood‐feeding with significant skin damage and bleeding of capillaries. This aggressive feeding behaviour may provide a direct route to the blood stream for these motile bartonellae. In contrast, bartonellae encoding the VirB/VirD4‐T4SS (i.e., L3, L4, and *B. ancashensis*) have been primarily associated with transmission by lice and fleas. These arthropods display a more subtle blood‐feeding behaviour that does not lead to capillary damage and thus not to a direct route for the bacteria to enter the blood stream. Rather, these bartonellae are superficially inoculated into the dermis by scratching and biting (Dehio & Tsolis, 2017; Siamer & Dehio, 2015), which necessitates that bacteria colonise additional host niches before reaching the blood. We therefore hypothesize that the independent acquisition of the VirB/VirD4‐T4SS in *B. ancashensis* and the last common ancestors of L3 and L4, represents an adaptive trait and evolutionary key innovation that provided novel ecological opportunities to the bartonellae, that is, vector competence for arthropods with bacterial transmission relying on systematic spread of infection from a dermal site of inoculation. Host adaptation by expanding and diversifying Bep repertoires has then driven the parallel evolutionary trajectories of explosive radiations seen in L3 and L4 (see below).

## 5 PARALLEL EVOLUTION OF COMPLEX BEP REPERTOIRES

**Figure 2:**
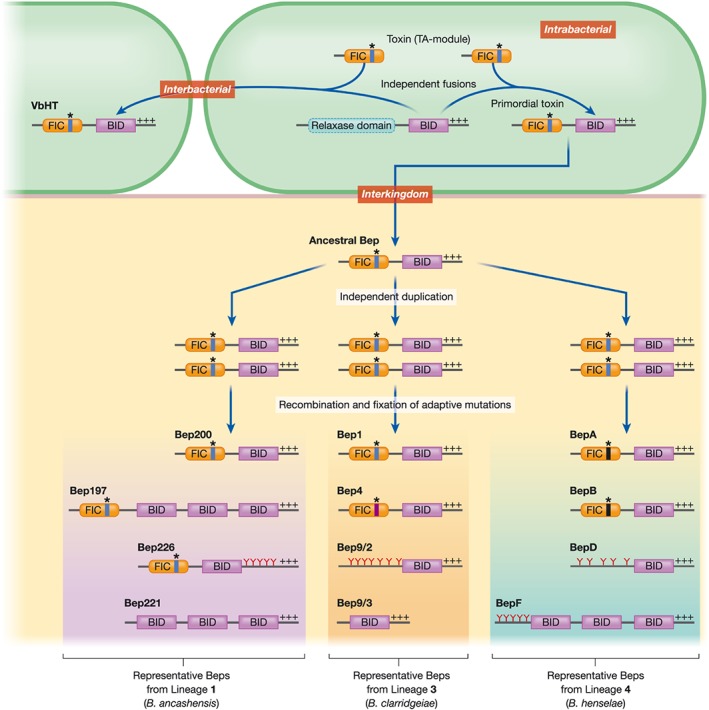
Parallel evolution of VbhT and Bep repertoires. Independent fusion of FIC‐domains (from intrabacterial toxin‐antitoxin (TA) modules) to a relaxase derived type‐IV‐secretion (T4S) signal leading to VbhT and a primordial toxin. This T4S signal is composed of a C‐terminal BID domain and a positive tail (+++).The primordial, interbacterial toxin evolved to an interkingdom effector—the ancestral Bep. The three Bep repertoires of *Bartonella ancashensis* (L1), L3, and L4 likely evolved from this ancestral Bep independently via gene duplication, followed by recombination and fixation of adaptive mutations. The majority of Beps (and VbhT) possess the FIC‐BID architecture; however, Beps with a derived domain composition evolved in all three lineages. Indicated are the catalytic FIC‐motif (*): conserved and canonical (HPFX[D/E]GNGRXXR; blue vertical line), conserved, but not canonical (CPFX[G/A]GNECTQX for Bep4 orthologues; purple vertical line) and not conserved (XPFXXGNXXTXX; for BepA orthologues, black vertical line) among orthologues. Tyrosine‐phosphorylation motifs are highlighted with an Y

Beps are multidomain proteins composed of an N‐terminal effector domain and a C‐terminal bipartite T4S signal (Engel et al., 2011; Siamer & Dehio, 2015). The most common effector domain is Filamentation induced by cyclic‐AMP (FIC) that mediates post‐translational modifications (PTMs) of target proteins (Harms, Stanger, & Dehio, 2016). The bipartite T4S signal is composed of a Bep intracellular delivery (BID) domain and a C‐terminal stretch enriched for positively charged residues (Schulein et al., 2005). The C‐terminal BID domains are crucial for translocation and considered to interact directly with the T4CP. Genome analysis revealed that 70% of all Beps display the canonical FIC‐BID architecture (Engel et al., 2011). The remaining Beps, however, lack a FIC domain and instead harbour tandem‐repeated tyrosine (pY)‐motifs and/or additional BID domains (Figure 2).

### 5.1 The FIC domain

The FIC domain is found in proteins of all domains of life, including intrabacterial effectors such as the ubiquitous FicT toxins of toxin‐antitoxin (TA) modules, interbacterial effectors such as VbhT, and interkingdom effectors translocated by various bacterial secretion systems into mammalian host cells (Harms et al., 2015). The enzymatic activity of FIC domains typically catalyses transfers of an AMP moiety (AMPylation) onto a hydroxyl‐group side‐chain of target proteins, thereby modulating cellular physiology. This covalent modification typically interferes with the cellular function of the target protein, such as shown for AMPylation of small GTPases that interferes with downstream signalling via blocking the interaction with physiological effectors (Harms et al., 2016). AMPylation relies on a conserved FIC signature motif that is part of the enzymatic cavity of the FIC protein. Of note, many FIC proteins share non‐canonical FIC signature motifs and thus might catalyse different PTMs. A notable example constitutes the *Legionella* effector AnkX, which phosphocholinates the small GTPase Rab1 (Mukherjee et al., 2011).

In *Bartonella*, the interbacterial effector VbhT mediates AMPylation of the bacterial type II topoisomerases gyrase and topoIV, resulting in their inactivation (Harms et al., 2015). Among the FIC domain‐containing Beps, AMPylation activity was demonstrated for the L4 effector BepA from *B. henselae* and the L3 effector Bep2 from *B. rochalimae* (Palanivelu et al., 2011; Pieles, Glatter, Harms, Schmidt, & Dehio, 2014). Orthologues of the L3 effectors Bep1, Bep2, and Bep3 and the FIC domain‐containing Beps of *B. ancashensis* (L1) display a conserved FIC signature motif, indicating that they may modulate host cellular function by AMPylation (Figure 2). Other orthologous effectors (such as Bep4) display non‐canonical FIC signature motifs that are, however, conserved among the orthologues, suggesting that they may contribute to the infection process by conferring PTMs different to AMPylation. Finally, the Fic domains of some orthologous groups (e.g., BepA/B and Bep5) are not conserved, which might be indicative for a loss of enzymatic activity, or for an enzymatic switch confined to sub‐lineages (Harms, Segers, et al., 2017b).

### 5.2 Tandem‐repeated pY‐motifs

Bacterial pathogens selectively manipulate mammalian signalling processes by translocating effectors harbouring tandem‐repeated pY‐motifs, which mimic eukaryotic host proteins. Host cellular kinases phosphorylate these pY‐containing effectors, which subsequently interact with SH2 domain proteins (Selbach et al., 2009). Two pY‐Beps, *B. henselae* BepD and BepE, have been shown to recruit SH2 domain‐containing proteins following phosphorylation mediated by the host tyrosine kinase c‐Src (Schulein et al., 2005; Selbach et al., 2009). Although their specific biological function remains to be demonstrated, the conservation of BepD/BepE within L4 Bartonellae suggests an important role in host cell manipulation. Intriguingly, the presence and conservation of pY‐Beps in L1 (Bep226), L3 (Bep9/2), and L4 (BepD/E/F/H; Figure 2) suggests that (a) pY‐motifs evolved de novo in all three lineages, followed by their tandem duplication and diversification, and (b) that these effectors play a fundamental role in host cell manipulation.

### 5.3 The BID domain

As part of the C‐terminal bipartite T4S‐signal, the BID domain is present in all Beps (Schulein et al., 2005; Harms, Segers, et al., 2017b). As the result of several gene duplication events, some Beps (e.g., L1:Bep197, Bep211; L4: BepE/F/G) harbour multiple copies of BID domains (Figure 2). These non‐terminal BID domains are likely released from selective pressure to interact with the T4CP and are thus free to adopt novel functions. In fact, BID domains contribute to most of the Bep‐mediated effector functions in host cells that are currently known. For instance, the BID domains of BepF/BepG trigger F‐actin‐dependent uptake of *B. henselae* into endothelial cells (ECs; Rhomberg, Truttmann, Guye, Ellner, & Dehio, 2009; Truttmann, Guye, & Dehio, 2011). Furthermore, it has been shown that the two BID domains of BepE are required for normal host cell migration during infection (Okujava et al., 2014). The best understood example of a BID domain affecting host cellular functions is the inhibition of apoptosis mediated by BepA from *B. henselae* (5). The single BID domain of BepA has been shown to (a) mediate BepA translocation into ECs, and (b) to interact directly with human adenylyl cyclase to stimulate cyclic‐AMP production and consequently the inhibition of apoptosis (Pulliainen et al., 2012).

The structures of three different BID domains revealed a novel, conserved fold formed by a four‐helix bundle topped with a hook (Stanger et al., 2017). Although the core of the BID domain is formed by conserved apolar residues, the surface of the BID domain reveals a high degree of variability even among orthologues. On the basis of the solved BID domain structures, the conserved overall fold suggests a crucial role in initial steps of T4SS mediated Bep translocation into host cells. In contrast, the less conserved surface of BID domains seems to have facilitated the evolution of new interaction interfaces with host target proteins, thereby modulating different cellular pathways (Stanger et al., 2017). We further believe that the high degree of surface variability of BID domains of Bep orthologues might display an adaptive step to fine‐tune host‐restricted interactions. This idea is supported by the fact that apoptosis inhibition of human ECs can be triggered by BepA from zoonotic *B. henselae* and human‐specific *B. quintana*, but not by BepA from the rat pathogen *B. tribocorum* (Schmid et al., 2006).

Although the sequence divergence of individual domains within canonical FIC‐BID Beps is indicative of functional specification, the multidomain architecture of some Beps might also contribute to the functional plasticity of these effectors.

### 5.4 Parallel evolution of Bep repertoires from a primordial bacterial toxin

The Bep arsenals found in *B. ancashensis* (L1) and in species of L3 and L4 arose three times through independent duplication events from a single, primordial FIC‐BID effector, followed by a cascade of gene duplication and diversification events (Figure 2; Siamer & Dehio, 2015; Dehio & Tsolis, 2017; Harms, Liesch, et al., 2017a). Initially, it was proposed that the FIC‐BID‐toxin VbhT represents a missing link in the evolution of Beps (Siamer & Dehio, 2015). A recent phylogenetic analysis challenged this hypothesis by showing that the Fic domains of VbhT and of the Beps are phylogenetically distinct. Thus, the Fic domains of VbhT and a primordial toxin must have independently fused to a BID domain derived from a relaxase involved in interbacterial conjugation. When co‐opting VirB/VirD4‐T4SS for host interaction the interbacterial primordial toxin evolved into an interkingdom effector—the ancestral Bep. Independent acquisitions of this ancestral Bep by three eubartonellae lineages followed by repeated rounds of duplication and diversification events and fixation of adaptive mutations then led to the emergence of complex Bep repertoires present in modern eubartonellae. Thus, it appears that VbhT and the three Bep arsenals, respectively, are the result of parallel evolution (Harms, Liesch, et al., 2017a; Figure 2). Fusion events of enzymatic domains to type‐IV‐secretion domains have occurred more frequently, suggesting that the de novo creation of secreted, interbacterial toxins via T4SSs represents a key step in the evolution of interkingdom effectors (Harms, Liesch, et al., 2017a).

## 6 THE *BARTONELLA* GENE TRANSFER AGENT—THE DRIVING FORCE OF *BARTONELLA* EVOLUTION

The *Bartonella* specific gene transfer agent (BaGTA) is highly conserved within the genus *Bartonella* (Figure 1) and considered crucial for genome integrity and adaptive evolution (Berglund et al., 2009). A subset of homologous BaGTA genes is also present in the ant‐associated species *Candidatus* Tokpelaia hoelldoblerii, suggesting that the domestication of the BaGTA began at least at the onset of the Bartonellaceae (Tamarit, Neuvonen, Engel, Guy, & Andersson, 2018). Genomically linked to the *BaGTA* gene cluster is a region of high plasticity, which harbours diverse virulence factors, including, for instance, the VirB/VirD4‐T4SS and its respective Beps. The BaGTA has been early on proposed to be instrumental for the independent acquisitions of the distinct T4SSs and gene duplications and thus for the explosive radiation observed within the eubartonellae (Guy et al., 2013). Experimental evidence with *B. henselae* confirmed the role of the BaGTA in gene transfer and revealed an unexpected preference for the fittest bacterial subpopulation in contributing preferentially as donors and recipients of gene transfer (Guy et al., 2013; Quebatte et al., 2017). It remains to be demonstrated at what stage of the *Bartonella* life cycle BaGTA transfer occurs preferentially. The arthropod midgut that is often colonised by diverse *Bartonella* strains (Chomel et al., 2009) may provide the best ecological opportunity for this highly efficient gene transfer to occur (Quebatte et al., 2017). However, while the role of the BaGTA in genome integrity and adaptive evolution is highly appreciated, it is not clear whether the BaGTA plays also a direct role in pathogenesis. Interestingly, it was recently shown that transposon‐mutants with insertion at different sites of the *BaGTA* locus showed impaired induction of GFP under the control of the *virB*‐promoter, suggesting a role of the BaGTA in VirB/VirD4‐T4SS expression (Quebatte, Dick, Kaever, Schmidt, & Dehio, 2013). Future work will be required to elucidate at which stage of the infection cycle the BaGTA is active and if it plays a direct role during pathogenicity within the mammalian host.

## 7 CONCLUDING REMARKS

Recent findings have greatly advanced our understanding of the virulence mechanisms underlying the remarkable evolutionary and ecological success of pathogens within the genus *Bartonella*, which led to the emergence of a large number of species each adapted to cause highly prevalent infection in their specific mammalian reservoir host. T4SSs represent evolutionary key innovations for host adaptation of the bartonellae and have been horizontally acquired multiple times by different lineages. T4SSs are crucial for species‐specific erythrocyte infection (Trw‐T4SS) and for translocation of Bep effectors to subvert host cellular processes (VirB/VirD4‐T4SS). Although functional analyses of selected Beps have yielded first insights in their molecular mode of function, future studies should aim at systematically identifying host targets for all Beps and decipher their underlying molecular mechanisms of manipulating specific host cellular functions. A more far‐reaching goal will then be to unravel how the individual molecular activities of the various Beps that are co‐injected into host cells are orchestrated in space and time to benefit the infection process. Moreover, the high variability of Bep orthologues among closely related species is indicative for host adaptation within their specific reservoirs. Comparative analyses of Bep‐target interactions in the evolved pathogen‐host pairs will then allow addressing the proposed role of Beps in mediating host specificity. Finally, the three Bep arsenals that evolved independent from a primordial Bep in three *Bartonella* lineages will facilitate studying parallel trajectories of convergent and divergent evolution in this remarkable example of host adaptation.
